# Risk Factors and Prevention Strategies for Complex Regional Pain Syndrome Following Fractures: A Scoping Review

**DOI:** 10.7759/cureus.109118

**Published:** 2026-05-18

**Authors:** Tina Tran, Brian H Tran, Tatum R Dam, Ank A Agarwal, Moorice Caparó

**Affiliations:** 1 College of Osteopathic Medicine, Kansas City University, Joplin, USA; 2 Department of Biomedical Data Science, Stanford University School of Medicine, Stanford, USA; 3 Department of Physical Medicine and Rehabilitation, Montefiore Hutchinson Campus, Bronx, USA

**Keywords:** complex regional pain syndrome, fracture, prevention, risk-factors, scoping review

## Abstract

Complex regional pain syndrome (CRPS) is a neuropathic pain disorder characterized by pain disproportionate to the severity of the inciting injury, persisting beyond the expected period of tissue healing. CRPS can significantly impact functional outcomes, but despite increasing research, risk factors and preventive strategies remain incompletely characterized. The objective is to map the existing evidence on risk factors and preventive strategies for CRPS following fracture, including trauma-related, surgical, and patient-related contributors, and identify gaps in current prevention and risk stratification approaches. A comprehensive search of PubMed, Embase, and Scopus was conducted from database inception through March 17, 2026. Eligible studies included human studies of any design evaluating CRPS risk factors or prevention following fracture. Two independent reviewers performed screening and data extraction. Data was synthesized narratively. Thirty-one studies were included. Preventive strategies most involved early mobilization and vitamin C supplementation, though evidence for vitamin C was heterogeneous. Limited evidence suggested potential benefit from aspirin and N-acetylcysteine. Female sex, psychiatric comorbidities, high pain intensity, and fracture severity were the most consistently reported risk factors. Evidence was largely derived from observational studies. Evidence supporting prevention strategies for CRPS remains limited and heterogeneous. Early mobilization appears beneficial, while pharmacologic strategies require further evaluation and validation. Identified risk factors highlight the multifactorial nature of CRPS and underscore the need for validated risk stratification tools and high-quality prospective studies.

## Introduction and background

Complex regional pain syndrome (CRPS) is a neuropathic pain disorder characterized by pain disproportionate to the severity of the inciting injury, persisting beyond the expected period of tissue healing. It arises most commonly after a fracture, though surgeries, sprains, contusions, and crush injuries are also frequent precipitants; in some cases, prolonged immobilization or no identifiable injury precedes the syndrome [1]. Epidemiologic studies consistently identify fractures as the most common precipitating event, with one large retrospective cohort study reporting fractures as the cause of 44% of CRPS cases [2]. Upper extremity injuries in middle-aged women represent the most affected demographic, with distal radius fractures, commonly known as Colles fractures, accounting for up to 36.7% of cases in some studies [1]. Additional fracture-related risk factors include intra-articular fractures, dislocations, comminuted injuries, and prolonged casting, all of which may further predispose patients to the development of this condition [3].

CRPS is classified into two subtypes. Type I, or reflex sympathetic dystrophy, occurs without a documented nerve injury and accounts for approximately 90% of cases. Type II, or causalgia, requires a known associated nerve lesion. Clinically, the subtypes are indistinguishable: both follow a regional distribution rather than a specific dermatomal or peripheral nerve territory, and symptoms predominantly involve the distal extremities, though proximal or contralateral spread may occur [4].

Classic features include allodynia (pain elicited by typically nonpainful stimuli) and hyperalgesia (an exaggerated response to normally painful stimuli). Autonomic changes include alterations in skin color, temperature, and sweating. Examination may reveal weakness, reduced range of motion, tremor, and dystonia. The pain does not conform to a dermatomal or myotomal distribution.

No widely accepted pathophysiological mechanism has been established, and no gold-standard diagnostic test exists. Diagnosis rests on clinical evaluation using the Budapest criteria, which requires ongoing pain that is disproportionate to the inciting event, symptoms across multiple sensory, vasomotor, sudomotor/edema, and motor/trophic domains with corresponding objective examination findings, and exclusion of alternative diagnoses that better explain the presentation [1].

CRPS is a debilitating condition that impairs function, sleep, and daily activities and carries a substantial psychological and social burden. Delayed intervention is associated with worse outcomes, whereas early-stage disease is more responsive to treatment. Treatment goals include alleviating pain, restoring previous function, and preventing long-term disability [4]. Management requires a multidisciplinary approach encompassing physical and educational therapy, pharmacotherapy--bisphosphonates, glucocorticoids, and vasoactive agents appear most effective, and interventional strategies such as sympathetic nerve blocks [1,4].

Despite growing research into CRPS, prevention strategies and risk factors remain relatively underexplored, with much of the current evidence fragmented and heterogeneous. This scoping review aims to map the existing evidence on risk factors and preventive strategies for CRPS following fractures, including trauma-related, surgical, and patient-related contributors, and identify gaps in current prevention and risk stratification approaches.

## Review

Methods

This scoping review was conducted in accordance with the methodological framework described by Arksey and O'Malley [5] and further refined by Levac et al. [6]. Reporting follows the Preferred Reporting Items for Systematic Reviews and Meta-Analyses Extension for Scoping Reviews (PRISMA-ScR). No formal protocol was registered for this scoping review.

Search Strategy

A comprehensive literature search was conducted in PubMed, Scopus, and Embase from database inception through March 17, 2026. The search strategy combined keywords and controlled vocabulary (e.g., MeSH and Emtree terms where applicable) and keyword combinations related to CRPS and fractures. Boolean operators (AND/OR) were used to combine search concepts, and search strategies were adapted for each database.

The search strategy combined keywords and controlled vocabulary related to CRPS and fracture, including: “complex regional pain syndrome,” “CRPS,” “fracture,” “distal radius,” “immobilization,” “early mobilization,” “risk factors,” and “prevention."

A sample PubMed search strategy was as follows: (“complex regional pain syndrome” OR “CRPS”) AND (“fracture” OR “distal radius fracture”) AND (“risk factors” OR “prevention” OR “immobilization” OR “rehabilitation”).

Search strategies were adapted for each database. Reference lists of included studies were also screened to identify additional relevant articles.

Inclusion and Exclusion Criteria

Eligible studies included human studies of any design--randomized controlled trials, cohort studies, case series, and case reports--that evaluated CRPS after trauma or fracture or assessed prevention methods and risk factors. All study designs were eligible to comprehensively map the breadth of available evidence, consistent with scoping review methodology. Non-English articles, review articles, animal studies, and studies addressing CRPS treatment without discussion of risk or prevention were excluded.

Critical Appraisal of Individual Sources of Evidence

Consistent with scoping review methodology and PRISMA-ScR recommendations, a formal critical appraisal of study quality was not performed, as the objective was to map the extent and nature of available evidence rather than to assess risk of bias or evaluate study quality. Due to this, findings should be interpreted as descriptive of the available literature, not as an assessment of the overall quality or strength of evidence. By the same token, the descriptive percentages included throughout the Results section reflect the proportion of included studies identifying specific findings rather than pooled patient-level estimates. A meta-analysis was not performed; thus, these frequencies should be interpreted as descriptive measures of trends within the literature rather than the strength and quality of association.

Study Selection and Data Extraction

**Figure 1:**
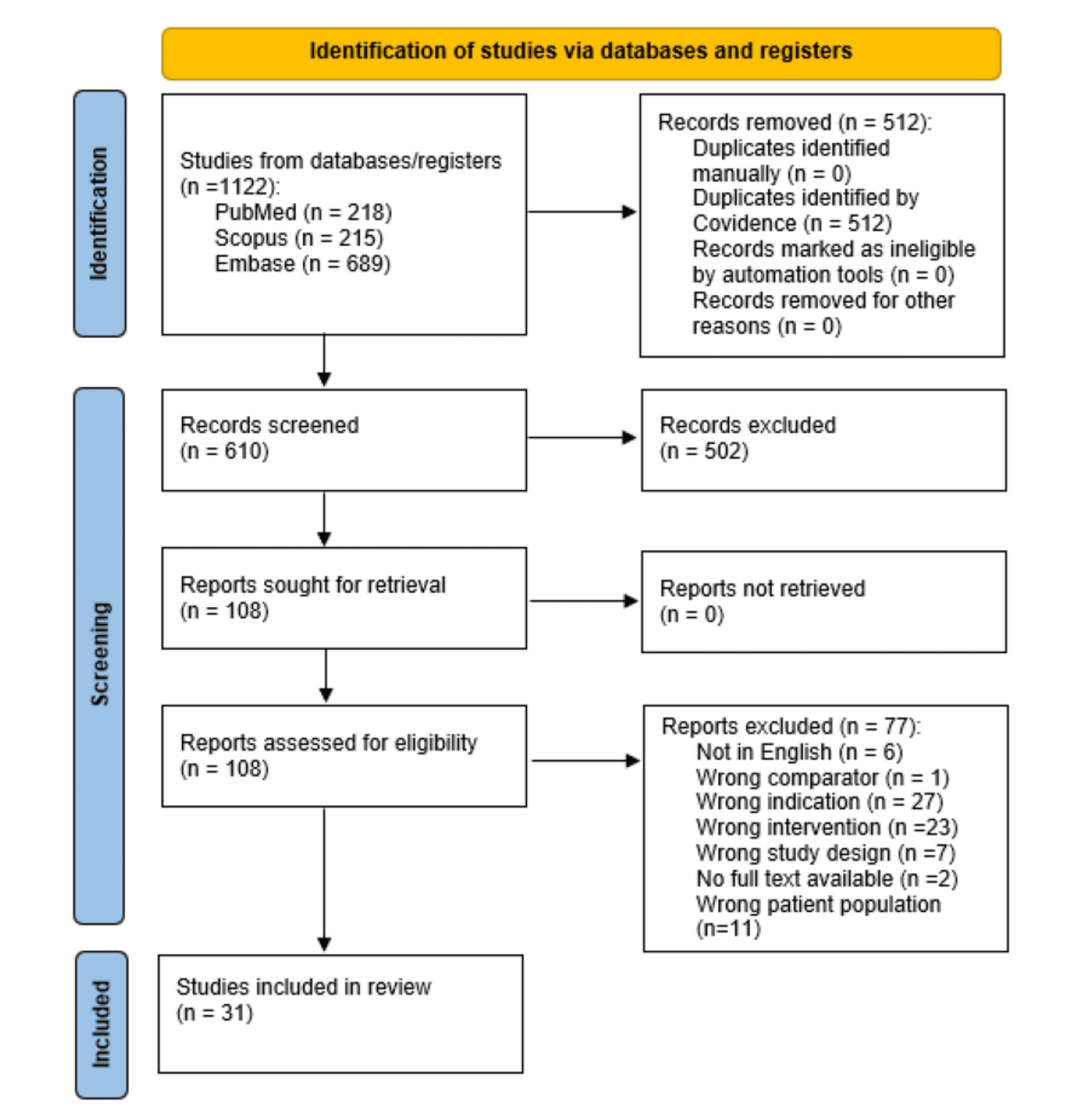
PRISMA 2020 flow diagram of study selection

Two independent reviewers screened titles and abstracts using Covidence software. Prior to formal screening, eligibility criteria were discussed and refined by reviewers to promote consistent study selection. Discrepancies were resolved through discussion and consensus. Full-text review was performed for all potentially eligible studies. A standardized data extraction template was developed by reviewers and piloted in the initial stages of extraction to assess if refinement was necessary. The template was determined to adequately capture the relevant study variables. Data extraction was performed independently by reviewers, with discrepancies resolved by consensus. Numerical data for the study selection is included in the PRISMA diagram (Figure 1).

Data Items

Data extracted included author, year, study population, risk factors identified, preventive methods assessed, injury type, and outcome. Where necessary, study findings were grouped into conceptual categories (e.g., demographic, clinical, and injury-related risk factors) to facilitate synthesis.

Results

Synthesis of Results 

Where appropriate, study findings were summarized and organized into broader conceptual categories such as demographic, clinical, and injury-related factors as well as preventive interventions. These findings were then reviewed to identify recurring themes and areas of consistency or variation across studies.

Search Yield 

The initial search yielded 610 studies meeting preliminary eligibility criteria. Following title and abstract screening, 108 articles underwent full-text review, and 31 studies were ultimately included for thematic synthesis based on relevance to CRPS risk factors and prevention. Reasons for exclusion at the full-text stage included studies not evaluating CRPS outcomes, lack of fracture-related populations, and absence of relevant risk or prevention data.

Study Characteristics

Of the 31 included studies, 20 examined distal radius fractures, 5 addressed non-specific upper and lower extremity injuries, 5 focused on hand and wrist injuries, and 1 evaluated foot and ankle injuries.

**Table 1:** Preventive strategies for complex regional pain syndrome following fractures Abbreviations: CRPS, complex regional pain syndrome; RCT, randomized controlled trial

Study (reference)	Study Design	Fracture Type	Preventive Measure	Outcome
Zincirci 2026 [7]	Randomized controlled trial (RCT)	Distal radius fracture	Contralateral isokinetic wrist strengthening during immobilization	Reduced likelihood of CRPS
Baygutalp 2020 [8]	Retrospective cohort study	Proximal and distal upper and lower extremity fractures	Early rehabilitation	Reduced likelihood of CRPS
Boersma 2022 [9]	Prospective observational study (continuation of proof-of-concept cohort)	Distal radius fracture	Active treatment approach	Reduced likelihood of CRPS
Boersma 2018 [10]	Prospective proof-of-concept cohort study	Distal radius fracture	Home exercise program, plaster immobilization, and early mobilization	Reduced likelihood of CRPS
Alimian 2021 [11]	Randomized controlled trial (RCT)	Distal radius fracture	500 mg vitamin C as a Bier block adjuvant vs sterile water	Reduced likelihood of CRPS
Zollinger 2007 [12]	Double-blind, prospective, multicenter trial	Wrist fractures	200, 500, or 1500 mg of vitamin C daily for 50 days	Reduced likelihood of CRPS
Besse 2009 [13]	Prospective randomized controlled trial	Foot and ankle surgery	1 g daily vitamin C treatment	Reduced likelihood of CRPS
Ekrol 2014 [14]	Double-blind, randomized controlled trial	Displaced or nondisplaced distal radius fracture	500 mg of vitamin C	No statistically significant benefit
Dinç 2024 [15]	Retrospective, single-center study	Distal radius fracture	N-acetylcysteine (NAC)	Reduced likelihood of CRPS
Eraghi 2020 [16]	Randomized controlled trial	Closed, unilateral, extra-articular distal radius fracture	Aspirin	Reduced likelihood of CRPS
Cowell 2018 [17]	Quality improvement/implementation study	Distal radius fracture	Multimodal practice intervention (patient education, standardized care protocol, staff education, visual aids)	Reduced likelihood of CRPS
Zlatkovic-Svenda 2019 [18]	Randomized controlled trial	Distal radius fracture	Bioptron light therapy combined with conventional therapy	Reduced likelihood of CRPS

**Table 2:** Risk factors associated with complex regional pain syndrome following fractures Abbreviations: ORIF, open reduction and internal fixation; VAS, visual analog scale; DASH, Disabilities of the Arm, Shoulder and Hand; GAD-7, Generalized Anxiety Disorder-7; PHQ-9, Patient Health Questionnaire-9

Study (reference)	Study Design	Fracture Type	Risk Factors
Gong 2022 [19]	Multicenter, prospective observational study	Scaphoid waist fracture	Female sex, diabetes, and severe pain were reported before treatment
Roh 2014 [20]	Retrospective cohort study	Distal radius fracture	High-energy injuries, severe fractures, and female sex
Jellad 2014 [21]	Prospective study	Distal radius fracture	Female sex, trauma of low and medium energy, physical component summary score < 40, and PRWE pain subscale score >16
Jo 2019 [22]	Retrospective database cohort study	Distal radius fracture	Psychiatric factors, rheumatoid arthritis, ulnar fractures, open fractures, open reduction, and female sex
Jia 2022 [23]	Cross-sectional study	Radial head fractures treated with open reduction and internal fixation (ORIF)	Female sex, modified Mason type III fractures, and depressive personality disorders
Sumitani 2014 [24]	Retrospective cohort study	ORIF for limb fracture	Female sex, distal forearm, longer anesthesia duration
Demir 2010 [25]	Retrospective cohort study	Mechanical traumatic injury isolated to their hand or forearm	Motor nerve injury, female sex
Kaya 2025 [26]	Retrospective case–control study	Distal radius fracture	Older age, female sex, prolonged immobilization, and ACE inhibitor use
Xu 2024 [27]	Retrospective cohort study	Distal radius fracture	Older age, female sex, manual labor, complex fractures, internal fixation surgery, osteoporosis, elevated VAS (pain score), elevated DASH (functional disability score), and elevated GAD-7 (anxiety) and PHQ-9 (depression) scores
Pereira 2024 [28]	Retrospective cohort study	Upper extremity and lower extremity fractures	Preexisting anxiety disorders and mood disorders
Beerthuizen 2012 [29]	Prospective cohort study	Single fracture of the wrist, scaphoid, ankle, or metatarsal V	Intra-articular fractures, fracture dislocations, rheumatoid arthritis, musculoskeletal comorbidities, and increased baseline pain
Lipman 2019 [30]	Retrospective, observational cohort study	Distal radius fracture	Fibromyalgia
Dilek 2012 [31]	Prospective cohort study	Distal radius fracture	Anxious personality
Berk 2025 [32]	Retrospective cohort study	Upper extremity and lower extremity fractures	Vitamin D deficiency
Savaş 2018 [33]	Prospective cohort study	Traumatic hand injuries	Pain score of ≥ 5 in the first 3 days after surgery and crush injury
Herlyn 2010 [34]	Prospective cohort study	Distal radius fracture	rs1048101 single-nucleotide polymorphism within the α1a-adrenoceptor
Ratnasamy 2026 [35]	Retrospective cohort	Distal radius fracture	ORIF with concomitant carpal tunnel release
Farzad 2018 [36]	Prospective cohort study	Distal radius fracture	High pain intensity
Moseley 2014 [37]	Prospective cohort study	Wrist fracture	Increased baseline pain in the week after wrist fracture

Preventive Interventions

Increased movement and decreased immobilization time, particularly through physical therapy and exercise programs, were commonly reported preventive strategies for CRPS, with 33% (4/12) of studies highlighting its role in disuse and consequent muscle weakness prevention [7-10]. Beyond rehabilitation-based interventions, several studies also explored pharmacologic prevention methods. Vitamin C was among the most frequently examined agents due to its proposed antioxidant properties; however, current evidence demonstrated heterogeneity regarding benefit. Several studies reported an association between Vitamin C and decreased incidence of CRPS, including with oral supplementation and use during nerve blocks, while one study found no significant benefit [11-13].

Additional pharmacologic interventions were less frequently described but also suggested potential benefits. Two studies (16.7%) had found preventative benefit from alternative medications, including aspirin and N-acetylcysteine (NAC), though evidence remained limited with one study each [15,16]. Non-pharmacologic and educational interventions were comparatively underrepresented, with only one study describing the impact of practice changes such as patient information leaflets and education for staff regarding early warning signs [17]. Similarly, one study evaluated the benefit of polarized, polychromatic light therapy as another preventive modality [18].

Risk Factors

Identifying risk factors provides important insight into the prevention and management of CRPS. Across the included studies, female sex emerged as the most frequently reported demographic risk factor, identified in 9/19 (47.4%) of studies [19-27]. Older age was also identified as a demographic risk factor, although less consistently reported [26,27].

Comorbid conditions were also commonly associated with CRPS risk. Psychiatric conditions, particularly anxiety and depression, were among the most reported comorbidities, suggesting a potential biopsychosocial contribution, while musculoskeletal comorbidities were the second most reported [22,23,27-31]. Metabolic factors were less frequently examined, with 10.5% (2/19) of studies identifying associations with Vitamin D and diabetes [19,32].

Injury characteristics, including fracture type and location, were commonly identified as relevant risk factors for CRPS development [20,22-25,27,29,33]. Among the included studies, injury characteristics included high-energy or complex fractures (e.g., fracture dislocations, crush injuries, modified Mason type III fractures), and intra-articular fractures. Main locations included distal forearm and ulnar fractures [20,22-25,27,29,33]. Other factors, including genetic mutations, immobilization, angiotensin-converting enzyme (ACE) inhibitor use, and occupation, were reported less frequently, each supported by a single study, indicating emerging but limited evidence [26,27,34]. Functional status and treatment progression were also evaluated, with studies evaluating associations between functional ability scores and subsequent development of CRPS [20,27]. Perioperative factors, including type of surgery, anesthesia duration, and concurrent procedures, were also found to have a potential influence on CRPS risk [24,25,27,35]. Lastly, increased baseline pain was associated with increased incidence of CRPS across several studies [27,33,36,37].

Discussion

Despite growing research interest in CRPS, prevention strategies and risk factors remain incompletely characterized. Female sex, older age, and psychiatric or musculoskeletal comorbidities were the most consistently reported risk factors, while early mobilization and vitamin C supplementation were the most frequently studied preventive approaches. The evidence supporting each, however, is constrained by the methodological limitations described below.

Prevention Strategies

Within preclinical studies, vitamin C’s antioxidant properties have been hypothesized to reduce oxidative stress markers and inflammatory mediators observed within the skin, muscle, and nerve tissue of rat post-fracture models [38]. The reported efficacy of vitamin C remains heterogeneous within our search, and these results are consistent with the broader literature. While some randomized controlled trials and comparative studies suggest a significant reduction in CRPS incidence, others reported no statistically significant benefit and possibly even an increased incidence [39-42]. Therefore, the prophylactic potential of vitamin C warrants further investigation.

Early mobilization and reduced immobilization, such as avoiding delays in physical therapy after cast removal, emerged as key preventive CRPS strategies, which were also seen in broader literature [8,43]. Current evidence emphasizes that immobilization can induce several characteristics of CRPS, such as changes in temperature, mechanosensitivity, and thermosensitivity, and limb mobilization, is emphasized in current guidelines [44,45]. However, the data within this scoping review consisted of 3 observational studies and 1 randomized controlled trial consisting of 42 patients. Therefore, a larger-scale randomized controlled trial may be needed to increase the certainty of evidence.

Pharmacologic interventions, such as aspirin and N-acetylcysteine (NAC), were identified but were supported by limited evidence in our studies. These interventions demonstrate the potential benefit of anti-inflammatory and antioxidant pathways in CRPS prevention [46,47]. Specifically, NAC has been shown to reduce proinflammatory cytokines and oxidative stress markers in mouse models and as a relatively effective treatment for CRPS 1 in human trials [38,48]. With that said, aspirin has limited evidence in the current literature and has not been seen to significantly lead to a preventive effect [49]. Moreover, non-pharmacologic strategies, including patient education, staff recognition, and modalities such as polarized polychromatic light therapy, were also notably underrepresented despite their potential to facilitate early recognition and intervention. This gap highlights an important opportunity for future research.

Risk Factors

Regarding risk factors, injury characteristics, including fracture type and anatomic location, were reported in almost half of the included studies. High-energy and complex fractures (intra-articular fractures, fracture-dislocations, crush injuries, modified Mason type III fractures) as well as specified locations (distal forearm, ulnar fractures) appeared particularly associated with CRPS development. A more severe fracture has been seen with increased incidence of CRPS, possibly due to increased tissue damage and a greater pro-inflammatory response to the trauma compared to a simple fracture [50-52]. Additionally, fractures within the distal forearm and ulnar fractures are possibly correlated to an increased risk due to a similar pro-inflammatory response. These complex fractures may also lead to longer operation times, conferring a subsequent increase in anesthesia time, another risk factor for CRPS [24]. However, with these studies mainly observational, randomized controlled trials can allow for a more grounded theory.

This review identified several demographic and clinical factors associated with increased CRPS risk. Female sex emerged as the most consistently reported demographic risk factor. This is consistent with current studies that showed an increased incidence compared to males [2,44,53]. However, the underlying mechanisms remain unclear, with hypotheses including hormonal factors, genetic susceptibility, and other comorbidities such as asthma, migraines, and osteoporosis [54]. Moreover, older age was less frequently identified and may be a potential contributor, as seen in the broader literature [55,56]. Comorbid conditions, such as musculoskeletal disorders (rheumatoid arthritis) and psychiatric comorbidities (anxiety and depression), also appeared to play a significant role, supporting a possible biopsychosocial contribution to CRPS development [57]. Metabolic factors, including vitamin D deficiency and diabetes, were less commonly examined but may represent additional areas of interest.

Evidence for several additional risk factors was limited to isolated reports, including genetic factors, ACE inhibitor use, and occupational exposures. These findings should be interpreted cautiously and represent areas that require further validation studies.

*Gaps and Future Research Directions*

With the rarity of the condition, multiple gaps in the literature remain, and there is still no high-certainty evidence that supports the effectiveness of a particular therapy. More randomized controlled trials are needed to determine the efficacy of multimodal CRPS management strategies. However, it should also be noted that with such nuanced pathophysiological uncertainties, further research also needs to be implemented to identify individual variations and how these variations contribute to the overall presentation of CRPS. Moreover, additional research investigating potential mechanistic contributors, such as ischemia-reperfusion injury and tourniquet-related physiologic responses, may help clarify the pathways involved in CRPS development following fractures and surgical intervention.

Moreover, despite the identification of several potential risk factors, including older age, female sex, and higher initial pain intensity, the use of a validated and clinically applicable risk stratification tool has yet to be integrated into clinical practice. This highlights a critical gap between evidence and implementation that warrants further investigation since risk prediction models need reliable external validation before implementation into clinical practice.

Limitations

Several limitations should be considered when interpreting these findings. Available literature was limited by small sample sizes and heterogeneity in methodology, including differences in diagnostic criteria, follow-up duration, and outcome definitions. Moreover, many risk factors and preventive strategies were identified in a small number of studies, with several reported in only single investigations, limiting external validity and the strength of conclusions that can be drawn. Although this limitation may suggest underreporting within the literature rather than the absence of an association.

The predominance of observational and retrospective study designs further limits causal inference. Finally, as a scoping review, this study aims to map the available evidence, and since study quality was not formally assessed, potential publication bias may exist, which limits the strength of the interpretations and reported associations. These limitations highlight the need for additional studies with standardized definitions and prevention protocols to better inform CRPS risk stratification and prevention strategies.

## Conclusions

CRPS following fractures remains a condition with limited high-certainty evidence guiding prevention or risk stratification. Early mobilization and vitamin C supplementation are the most studied preventive approaches, though neither is supported by consistent, high-quality trial evidence. Female sex, psychiatric comorbidities, and fracture severity and location are the most reproducibly identified risk factors, pointing toward a biopsychosocial vulnerability that is not captured by injury characteristics alone. Standardized trials, coordinated outcome frameworks, and prospective validation of clinical risk models are needed to translate these associations into actionable prevention strategies.
